# Adverse effects and non-relapse mortality of BCMA directed T cell therapies in multiple myeloma: an FAERS database study

**DOI:** 10.1038/s41408-024-01023-9

**Published:** 2024-03-05

**Authors:** Zimu Gong, Godsfavour Umoru, Jorge Monge, Nishi Shah, Ghulam Rehman Mohyuddin, Sabarinath Venniyil Radhakrishnan, Rajshekhar Chakraborty, Leo Rasche, Carolina Schinke, Anita D’Souza, Meera Mohan

**Affiliations:** 1 Division of Hematology Oncology, Houston Methodist Hospital, Houston, TX USA; 2 Division of Hematology/Oncology, Weill Cornell Medicine, New York, NY USA; 3 Division of Hematological Malignancies, Department of Oncology, Montefiore Medical Center and Albert Einstein College of Medicine, New York, NY USA; 4 Division of Hematology/Oncology, University of Utah, Salt Lake City, UT USA; 5 Division of Hematology/Oncology, Department of Medicine, Medical College of Wisconsin, Milwaukee, Wisconsin USA; 6 Multiple Myeloma and Amyloidosis Program, Columbia University, Herbert Irving Comprehensive Cancer Center, New York, NY USA; 7 Department of Internal Medicine II, University Hospital of Würzburg, Würzburg, Germany; 8 Myeloma Center, University of Arkansas for Medical Science, Little Rock, AR USA

**Keywords:** Myeloma, Adverse effects

**Dear Editor**,

B-cell maturation antigen (BCMA)-directed T cell therapies such as idecabtagene vicleucel (ide-cel), ciltacabtagene autoleucel (cilta-cel), teclistamab, and elranatamab have changed the therapeutic landscape of patients with relapsed/refractory multiple myeloma (MM) [1–4]. Despite their effectiveness, chimeric antigen receptor (CAR) T-cell and bispecific antibody (bsAb) therapies have distinct toxicity profiles, including cytokine release syndrome (CRS), immune effector cell-associated neurotoxicity syndrome (ICANS), non-ICANS neurotoxicity, and the risk of infections, all contributing to significant morbidity and potentially non-relapse mortality (NRM) [1–11]. However, there is substantial variability in attributing treated-related adverse effects and mortality to the therapy in the pivotal registration clinical trials [12]. The U.S. Food and Drug Administration (FDA) Adverse Event Reporting System (FAERS) database contains reports of adverse events, medication error, and product quality complaints that were submitted to the FDA. Although healthcare providers and consumers voluntarily contribute to the data source, drug manufacturers are obligated to provide mandatory reporting. Herein, we analyzed the most reported adverse events and NRM among the FDA-approved BCMA-directed immunotherapy in MM.

For this study, we identified reports of adverse effects associated with ide-cel, cilta-cel, and teclistamab from the first quarter of 2019 to the second quarter of 2023. Elranatamab was not included in this analysis due to fewer reported cases (*n* = 23). Reporting odds ratio (ROR) was defined as odds of a reaction in a specific drug divided by the odds of that reaction in all other drugs. Non-relapse mortality was calculated by excluding disease progression from fatal cases. Odds ratio (OR) for mortality was defined as odds of fatal reports in a given drug divided by the odds of fatal reports in other drugs.

After excluding cases involving the use of more than one drug, a total of 1803 individual cases with 4423 adverse effects were identified. Table 1 summarizes the baseline characteristics of patients included. Overall, ide-cel (*n* = 584) and teclistamab (*n* = 723) had the most reported events. Adverse effects leading to hospitalizations were more common with teclistamab (53.5%) and cilta-cel (47.4%) compared to ide-cel (35.6%). Teclistamab demonstrated the highest rates of life-threatening events (*n* = 81; 11.3%) and death (*n* = 159; 22.1%) associated with an adverse effect among the 3 drugs (Table 1).

**Table 1:** Baseline characteristics and most reported adverse events in BCMA directed immunotherapy in patients with relapsed and/or refractory multiple myeloma.

Characteristic	Ide-cel	Cilta-cel	Teclistamab
Number of patients	584	477	723
Number of adverse events reported	2132	874	1363
Age- yrs. median (interquartile)	66 (58.5–71)	66 (57.8–71)	65 (57–72)
Female (%)^a^	225 (38.5)	117 (24.5)	229 (31.8)
Death (%)^a^	68 (11.6)	82 (17.2)	159 (22.1)
Life threatening (%)^a^	43 (7.4)	42 (8.8)	81 (11.3)
Hospitalization (%)^a^	208 (35.6)	226 (47.4)	385 (53.5)
CRS (%)^a^	344 (16.1)	90 (10.3)	130 (9.5)
ICANS (%)^a^	80 (3.8)	29 (3.3)	34 (2.5)
Non-ICANS neurotoxicity (%)^a^	215 (10.1)	56 (6.4)	52 (3.8)
Pneumonia (%)^a^	4 (0.2)	20 (2.3)	63 (4.6)
Sepsis (%)^a^	15 (0.7)	17 (2.0)	46 (3.4)
COVID-19 infection (%)^a^	4 (0.2)	24 (2.7)	69 (5.0)
Infection (%)^a^	4 (0.2)	2 (0.2)	19 (1.4)

Per 21CFR314.80, Life threatening event are defined as any adverse drug experience that places the patient, in the view of the initial reporter, at immediate risk of death from the adverse drug experience as it occurred, i.e., it does not include an adverse drug experience that, had it occurred in a more severe form, might have caused death.

^a^*N* (%)

Next, we specifically investigated the adverse effects of interest, namely CRS, ICANS, non-ICANS neurotoxicity, and infections. The rate of CRS was highest with ide-cel (16.1%), while the reported instances of ICANS were similar between ide-cel (3.8%) and cilta-cel (3.3%). Non-ICANS neurotoxicity was reported with both ide-cel (*n* = 215,10.1%) and cilta-cel (*n* = 56, 6.4%). Further, we looked into the most commonly reported attributes of non-ICANS neurotoxicity associated with these agents. Bell’s palsy was reported almost exclusively with cilta-cel (*n* = 13, 1.5%), with 1 case reported with teclistamab. Parkinsonism was seen more frequently with cilta-cel (*n* = 7, 0.8%) compared to ide-cel (*n* = 4, 0.2%). Infections such as pneumonia (*n* = 63; 4.6%), sepsis (*n* = 33; 2.4%) and COVID-19 (*n* = 39; 2.8%) were more common with teclistamab. Pneumocystis jirovecii pneumonia (*n* = 10, 0.7%), cytomegalovirus (CMV) reactivation (*n* = 9, 0.7%), and CMV pneumonia (*n* = 6, 0.4%) were also predominantly linked to teclistamab. Ide-cel was associated with 3 cases of multifocal leukoencephalopathy (PML), while none were reported with cilta-cel or teclistamab.

Next, we analyzed ROR for the most frequently reported adverse effects, namely CRS, ICANS, non-ICANS neurotoxicity and infection (Fig. 1A). Ide-cel exhibited the highest ROR for CRS (1.8, 95% CI 1.5–2.16) and non-ICANS neurotoxicity (2.19, 95% CI 1.73–2.77). Whereas for infection, cilta-cel showed a higher ROR (1.3, 95% CI 1.04–1.62) while ide-cel has lower ROR (0.13, 95% CI 0.1–0.17). Notably, teclistamab showed a highest ROR (4.38, 95% CI 3.61–5.31) for infections, but lowest ROR for CRS (0.63, 95% CI 0.51–0.78), ICANS (0.69, 95% CI 0.47–1.02) and non-ICANS neurotoxicity (0.4, 95% CI 0.3–0.54) in comparison to ide-cel and cilta-cel.

**Fig. 1:**
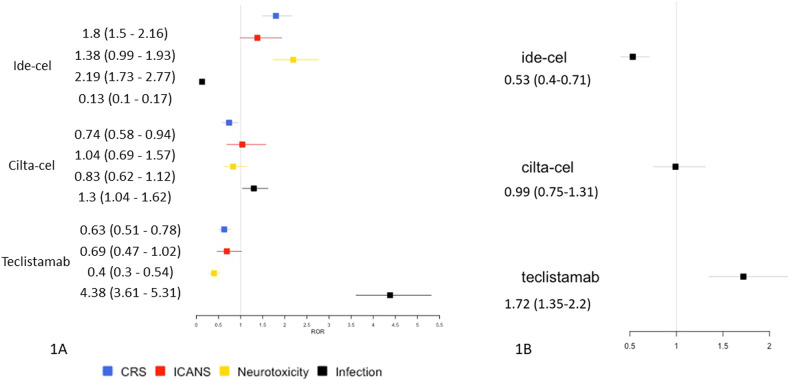
Forest Plots. **A** Forest plot of distribution of CRS, ICANS, Non-ICANS neurotoxicity and infection among BCMA directed immunotherapy. **B** Forest plot of the non-relapse mortality among BCMA directed immunotherapy in patients with relapsed and /or refractory multiple myeloma. ROR reporting odds ratio, OR odds ratio, NRM non relapse mortality.

We then analyzed the NRM attributed to these agents (Fig. 1B). The OR for NRM was compared between the 3 therapies and showed that ide-cel had the lowest OR (0.53, 95% CI 0.4-0.71) for NRM followed by cilta-cel (OR 0.99, 95% CI 0.75-1.31). Teclistamab exhibited the highest OR (1.72 95% CI 1.35–2.2) for NRM. The common events in NRM cases related to ide-cel were CRS, ICANS, and sepsis, whereas cilta-cel was associated with CRS, ICANS, and hemophagocytic lymphohistiocytosis (HLH). In NRM cases associated with teclistamab, the most prevalent events were infections including COVID-19, sepsis, and pneumonia.

Overall, our study highlights the distinctive toxicity profile associated with BCMA-directed T cell therapies in MM. We observe the highest rates of life-threatening events, hospitalization, and death with teclistamab compared to ide-cel and cilta-cel. Unlike CAR T-cell therapy with the current vein-to-vein time ranging between 47-71 days, bsAbs are readily available off-the-shelf options for patients with rapidly relapsing disease [13, 14]. Additionally, adequate organ function is prerequisite for CAR T-cell therapy, similar to the requirement for autologous stem cell transplant, inadvertently introducing a selection bias. The increased events and non-relapse mortality with teclistamab could be indicative of this patient population, or, alternatively, may be linked to significant morbidity and mortality linked to infections associated with bsAbs, as previously recognized [9].

The rates of CRS and ICANS were higher with BCMA CAR T-cell therapy compared to teclistamab. The spectrum of non-ICANS neurotoxicity associated with CAR T-cell therapy includes encephalopathy, tremor, aphasia, delirium, Parkinsonism, cranial nerve palsies, and peripheral neuropathies, like previously described occurrences [6]. Ide-cel has the highest ROR for non-ICANS neurotoxicity; however, this should be interpreted with caution due to a relatively larger number of ide-cel cases within the database, reflecting its status as the first FDA-approved BCMA CAR T-cell therapy. Parkinsonism was 4.3 times higher with cilta-cel compared to ide-cel. Bell’s palsy was exclusively observed with cilta-cel, with no reported cases associated with ide-cel. Both are consistent with clinical trial data [2].

Teclistamab was associated with a significantly stronger signal of infectious complication compared to ide-cel and cilta-cel, again consistent with current literature [9, 10]. The highest OR for NRM was noted with teclistamab followed by cilta-cel and ide-cel. While the exact attributes of NRM cannot be ascertained from the FAERS database, toxicities of CAR T-cell therapy and infections were the predominant events associated with NRM. Death unrelated to disease progression were reported with ide-cel in the KarMMa-1, KarMMa-3, and a real-world multi-institutional study and were attributed to complications such as CRS, HLH, neurotoxicity, COVID-19 infection, and cardiomyopathy [1, 8, 15]. The CARTITUDE 1 clinical trial also reported NRM attributed to various causes, including CRS/HLH, neurotoxicity, infections, and AML [2]. A recent study of commercial cilta-cel, reported a NRM of 9% (13/139) related to CRS, ICANS, delayed neurotoxicity and infections [14]. In the CARTITUDE 4 study of cilta-cel, 14% of deaths (25/39) among patients who received CAR T-cell infusion (*n* = 176) were attributed to causes unrelated to disease progression [5]. Similarly, clinical trial of bsAbs also reported cases of NRM related to COVID-19 infection, hepatic failure, and PML [3, 4]. Collectively, these findings reflect the substantial burden of NRM across various T-cell therapies.

Our analysis is limited by the nature of the FAERS database, where reporting is not mandatory, and selection bias cannot be eliminated. We presume consistent reporting practices for the same adverse events across different agents, ensuring the relevance of utilizing ROR for this analysis. Moreover, the timing of adverse effects, such as infection and NRM can be influenced by duration of treatment and time interval between events, and this data cannot be reliably calculated from the database. Nevertheless, since these agents share a similar indication for MM, clinicians will need to carefully consider their distinct toxicity profiles when choosing and sequencing treatments. Despite these limitations, these data offer a unique opportunity to comprehend adverse effects and NRM attributed to these novel therapies.

## Data Availability

The datasets generated during and/or analyzed during the current study are available from the corresponding author on reasonable request.
